# Clinical-molecular profiling of atypical *GNAO1* patients: Novel pathogenic variants, unusual manifestations, and severe molecular dysfunction

**DOI:** 10.1016/j.gendis.2025.101522

**Published:** 2025-01-09

**Authors:** Gonzalo P. Solis, Federica Rachele Danti, Yonika A. Larasati, Federica Graziola, Carolina Croci, Elisa Osanni, Alexey Koval, Giovanna Zorzi, Vladimir L. Katanaev

**Affiliations:** a Translational Research Center in Oncohaematology, Department of Cell Physiology and Metabolism, Faculty of Medicine, University of Geneva, Geneva 1211, Switzerland; b Child Neuropsychiatry Unit, Department of Pediatric Neuroscience, Fondazione IRCCS Istituto Neurologico Carlo Besta, Milano 20133, Italy; c Child Neuropsychiatry and Child and Adolescent Psychology, Department of Mental Health and Pathological Addiction, AUSL Piacenza, Piacenza 29121, Italy; d Scientific Institute, IRCCS E. Medea, Epilepsy and Clinical Neurophysiology Unit, Conegliano, Treviso 31015, Italy

G protein subunit alpha O1 (*GNAO1*)-related disorders represent a broad spectrum of neurological diseases mainly caused by *de novo* mutations in *GNAO1* encoding for G protein alpha subunit o (Gαo). As the major transducer of neuronal G protein-coupled receptors (GPCRs), Gαo is essential for the signaling involved in neuronal excitability and neurodevelopment. The most severe neomorphic *GNAO1*-mutations lead to developmental and epileptic encephalopathy-17 (DEE17; OMIM #615473) or neurodevelopmental disorder with involuntary movements (NEDIM; OMIM #617493), the latter with or without epileptic seizures.^1^ Movement disorders are present in almost all patients, with hypo/hyperkinetic features and profound impairment of postural development.^2^ Milder phenotypes including late-onset dystonia and parkinsonism with different extents of cognitive impairment have recently emerged from mutations leading to loss-of-function and haploinsufficiency.^3^ However, clear genotype–phenotype correlations and underlying pathogenic mechanisms are still poorly understood, limiting an accurate prediction of disease progression and the implementation of early therapeutic interventions. Here, we present two unrelated Italian patients carrying novel *GNAO1* mutations with atypical phenotypes, thus expanding the phenotypic spectrum of *GNAO1*-related disorders. We additionally provide a deep molecular analysis of the pathogenic Gαo variants along with a discussion of potential treatment options.

Patient 1 is a 13-year-old boy with severe intellectual disability, absence of speech, and autistic-like behavior without movement disorders (Video S1; Segment 1). His early development was marked by hypotonia and delayed milestones. At 6 months of age, he experienced a brief episode of hypotonia and ocular revulsion during a febrile episode, followed by two further seizures at 12 months of age, which were controlled with valproic acid. Brain magnetic resonance imaging was normal, but the electroencephalogram showed epileptic abnormalities. Patient 2 is a 16-month-old girl who showed apneic episodes shortly after birth, requiring short-term ventilatory support and phenobarbital therapy. She initially presented with hypotonia which progressed to subtle hypertonia and bradykinesia (Video S1; Segment 2 and 3). Her psychomotor development was broadly normal, with mild difficulties on scales assessing eye-hand coordination and personal-social-emotional skills. At present, neurological examinations revealed a mild rigid hypokinetic syndrome (Video S1; Segment 4 and 5); she remains seizure-free.

Supplementary video related to this article can be found at https://doi.org/10.1016/j.gendis.2025.101522

The following is the supplementary data related to this article:

Video S1 6 Video S1

The *de novo GNAO1* variants NM_020988.3:c.751T > C; p.Phe251Leu (F251L; patient 1) and c.791C > T; p.Ser264Phe (S264F; patient 2) were identified by next-generation sequencing and subsequently confirmed by Sanger sequencing. These variants have not been reported in the literature and are absent in the general population. *In silico* analysis suggests that both mutations are damaging/pathogenic (Table S1). Detailed clinical reports for both patients are found in supplementary data (summarized in Table S2A).

As with any G protein, Gαo cycles through the GDP (inactive) and GTP (active) states, which dictate its interactions with regulatory proteins and signaling effectors.^1^ To understand the underlying pathogenic mechanisms of the variants, we first determined their ability to incorporate and hydrolyze GTP using recombinant His_6_-Gαo wild-type, F251L, and S264F (Fig. S1A).^1^ F251L presented strong deviation from wild-type, showing an increase of ∼450% in GTP-uptake (k_bind_) and a ∼60% decrease in hydrolysis (k_hydr_; Fig. 1A–D). Agreeing with the mild patient phenotype, S264F showed only minor defects, with a small increase in k_bind_ and a decrease in k_hydr_ (Fig. 1E–H). We then analyzed their responses to ZnCl_2_ (the targeted drug currently in clinical applications to *GNAO1* patients^4^) and found that Zn^2+^ reduced GTP-uptake/hydrolysis by F251L in a concentration-dependent manner (Fig. 1I, J), while S264F was unaffected (Fig. S1B, C). Thus, F251L falls into class III, zinc-responsive category that includes G*NAO1* mutations associated with DEE17 and NEDIM, and S264F into class I involving zinc-unresponsive variants linked to milder phenotypes.^4^

**Figure 1:**
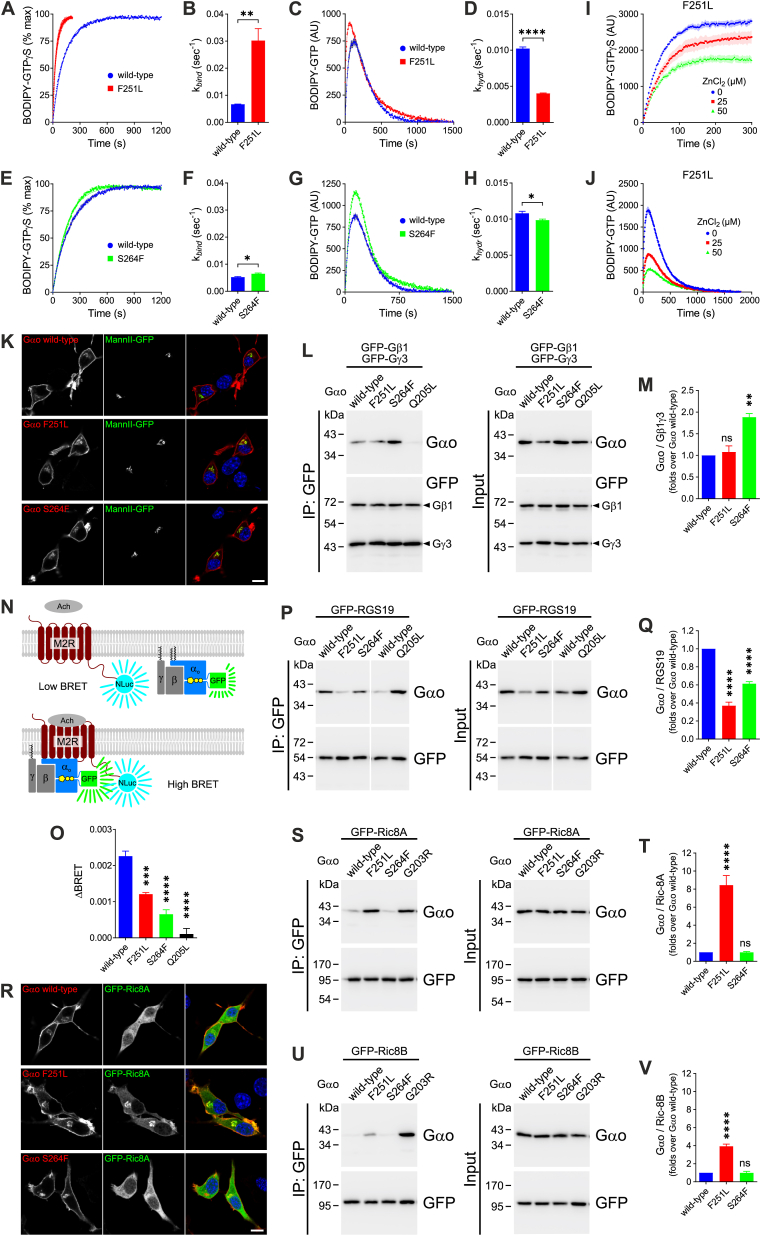
Biochemical, cellular, and structural characterization of the *GNAO1* variants c.751T > C; p.Phe251Leu (F251L) and c.791C > T; p.Ser264Phe (S264F). (A–H) The curves of the BODIPY-GTPγS uptake (A, E) and BODIPY-GTP hydrolysis (C, G) of recombinant His_6_-tagged Gαo wild-type together with the pathogenic F251L (A, C) or S264F (E, G) mutants, and quantification of the corresponding binding rate constants (k_*bind*_; *n* = 3 or 4) (B, F) and hydrolysis rate constants (k_*hydr*_; *n* = 3 or 4) (D, H). (I, J) The effect of increasing ZnCl_2_ concentrations on BODIPY-GTPγS binding (I) and BODIPY-GTP hydrolysis (J) by Gαo F251L. (K) Confocal images of N2a cells co-expressing Gαo wild-type or the pathogenic F251L and S264F mutants alongside a GFP-fusion of the Golgi marker mannosidase II (MannII-GFP). Cells were immunostained against Gαo and stained with DAPI in blue for nuclei. Scale bar, 10 μm. (L) HEK293T cells were co-transfected with GFP-tagged Gβ1 and Gγ3, and Gαo wild-type, F251L, S264F, or the constitutively active (non-pathogenic) Q205L mutant used as control. Immunoprecipitation (IP) of GFP-Gβ1γ3 was done using a nanobody against GFP, and the co-IP of Gαo proteins was analyzed by western blotting and immunodetection using antibodies against Gαo and GFP. (M) Quantification of the interaction between Gβ1γ3 and Gαo variants (*n* = 4). (N) An illustration of the BRET-based M2 muscarinic acetylcholine receptor (M2R)-coupling assay. M2R tagged with nano-luciferase (M2R-NLuc) excites the GFP-fusion of Gαo (Gαo-GFP). The steady-state low BRET signal increased upon acetylcholine (Ach) treatment (ΔBRET). (O) Quantification of the ΔBRET for Gαo wild-type, F251L, S264F, and Q205L (*n* = 3–6). (P) HEK293T cells were co-transfected with a GFP-tagged RGS19 construct, and Gαo wild-type, F251L, S264F, or the active Q205L control. The IP GFP-RGS19 and immunodetection were done as in (L). (Q) Quantification of the co-IP of Gαo constructs with RGS19 (*n* = 5). (R) Confocal images of N2a cells co-expressing a GFP-fusion of Ric8A (GFP-Ric8A) with Gαo wild-type or the pathogenic F251L and S264F variants. Cells were immunostained against Gαo and nuclei were stained in blue with DAPI. Scale bar, 10 μm. (S–V) HEK293T cells were co-transfected with the GFP-tagged Ric8A (S) or Ric8B (U) with Gαo wild-type, F251L, S264F, or the DEE17-linked G203R mutant used as control. IP and immunodetection were done as in (L). Quantification of the co-IP of Gαo variants and Ric8A (T) or Ric8B (V) (*n* = 4 or 5). Measurements are displayed as mean ± standard error of the mean. Data in (B, D, F, H) were analyzed by a two-sided unpaired *t*-test; for the remaining data, a one-way ANOVA followed by Dunnett's multiple comparisons test was used. ns, not significant; ∗*p* < 0.05, ∗∗*p* < 0.01, ∗∗∗*p* < 0.001, and ∗∗∗∗*p* < 0.0001.

At the cellular level, F251L and S264F presented a near-normal localization in N2a cells (Fig. 1K), characterized by plasma membrane and Golgi staining. Co-immunoprecipitation experiments from HEK293T cells showed normal Gβ1γ3 binding for F251L despite its reduced expression and a 1.9-fold increase for S264F (Fig. 1L, M; Fig. S2A). A higher formation of the heterotrimeric G protein by S264F was confirmed in the BRET (bioluminescence resonance energy transfer)-based Gβ3γ9 displacement assay, although it did not reach the level of the NEDIM-associated E246K variant (Fig. S2B, C).^1^ F251L, however, showed a ∼30% reduction in Gβ3γ9 association (Fig. S2C). Next, we examined Gαo engagement with the M2 muscarinic acetylcholine receptor in a BRET-based assay (Fig. 1N).^1^ Acetylcholine stimulation increased the BRET signal over basal (ΔBRET) by Gαo-GFP wild-type, a signal that dropped ∼50% for F251L and ∼70% for S264F (Fig. 1O). Poor GPCR coupling by the pathogenic Gαo variants was replicated in a BRET-based D2 dopamine receptor assay (Fig. S2D, E).

Most pathogenic Gαo mutants fail to interact with RGS (regulator of G protein signaling) proteins, probably due to folding defects that are key for the neomorphic Ric8 interactions.^1^ Following this trend, F251L and S264F showed a ∼60% and ∼40% reduction in RGS19 binding, respectively (Fig. 1P, Q). Like *GNAO1* mutations leading to DEE17/NEDIM,^1^ F251L induced a strong Golgi-relocalization of Ric8A, but S264F maintained normal cytoplasmic Ric8A (Fig. 1R). Co-expression of Ric8A normalized F251L expression level probably due to its strong interaction with Ric8A, whereas S264F showed a weak co-precipitation similar to Gαo wild-type (Fig. 1S, T; Fig. S3A). As neomorphic Ric8B interactions are particularly strong among DEE17 mutations,^1^ it was not surprising that F251L did not induce a robust Golgi-relocalization of Ric8B (Fig. S3B), and that it co-precipitated with Ric8B to a much lower extent than Gαo G203R, a DEE17-variant (Fig. 1U, V). S264F showed normal Ric8B localization and binding (Fig. 1U, V; Fig. S3B).

Finally, structure homology modeling showed F251L and S264F affecting the α3β5-loop adjacent to the switch III and C-terminal α5-helix domains, key regions in Gα-subunits for binding of downstream effectors and GPCR coupling, respectively (Fig. S4A). The Phe→Leu substitution disrupts the aromatic F251–F224 interaction in the active GTP-loaded Gαo structure, destabilizing the α3β5-loop/switch III interface (Fig. S4B). Predicted to interfere with Gαo adopting the active conformation, this probably causes the enzymatic defects and neomorphic Ric8 binding by F251L. On the other hand, S264 forms an H-bond with N346, stabilizing the α5-helix in the GDP-bound state (Fig. S4C). The Ser→Phe substitution likely induces the α5-helix misalignment, thus interfering with GPCR coupling (Fig. S4D). The remaining structure of the S264F seems unchanged, agreeing with its mild biochemical/cellular defects (see *Materials and Methods* in supplementary data for more details).

In summary, F251L presented strong GTP binding/hydrolysis defects, reduced GPCR coupling despite the near-normal formation of heterotrimeric G proteins, and gained a robust neomorphic binding to Ric8A and much weaker to Ric8B (Table S2B). These features place F251L on a par with NEDIM variants.^1^ In this regard, however, F251L appears unique among the Gαo variants described to date, as movement disorders are not present in our patient that only shows intellectual disability and lack of expressive speech common in NEDIM patients.^2^ Curiously, a pathogenic *GNAO1* c.980C > G; p.T327R mutation was reported associated with severe speech and intellectual disabilities but without movement disorders or seizures,^5^ and our ongoing analyses identify T327R as neomorphic for Ric8A with increased GTP-uptake (unpublished). Thus, these variants might represent a novel subgroup within the phenotypic spectrum. The zinc responsiveness of F251L may recommend patient inclusion in the clinical trial for oral zinc in *GNAO1*-related disorders (ZINCGNAO1, NCT06412653).^4^ For S264F, its suboptimal GPCR coupling predicts increased levels of inactive Gαo, which explains the higher Gβγ and lower RGS interactions. Together with the lack of neomorphic Ric8A/B binding, S264F appears as a partial loss-of-function for GPCR signaling, although it might also produce dominant-negative effects by sequestering Gβγ. Hence, S264F aligns with mutations at the milder end of the spectrum, similar to *GNAO1* c.644G > A; p.C215Y.^1^^,^^3^ Its lack of responsiveness to zinc makes the patient unlikely to respond to the treatment in the clinical setting.

Altogether, we described two patients with atypical clinical manifestations, thus expanding the phenotypic spectrum of *GNAO1*-related disorders, and additionally provided insights into the genotype–phenotype correlations.

## Ethics declaration

Clinical information was collected from medical reports; caregivers gave informed consent for the study and publication of the video and patient-related information. The study was approved by the territorial ethics committee (approval #CET 80/24).

## CRediT authorship contribution statement

**Gonzalo P. Solis:** Writing – review & editing, Writing – original draft, Methodology, Investigation, Formal analysis. **Federica Rachele Danti:** Writing – review & editing, Validation, Methodology, Investigation, Formal analysis, Data curation. **Yonika A. Larasati:** Writing – review & editing, Writing – original draft, Methodology, Investigation. **Federica Graziola:** Methodology, Investigation. **Carolina Croci:** Methodology, Investigation. **Elisa Osanni:** Methodology, Investigation. **Alexey Koval:** Methodology, Investigation, Formal analysis. **Giovanna Zorzi:** Writing – review & editing, Writing – original draft, Validation, Supervision, Project administration, Formal analysis, Conceptualization. **Vladimir L. Katanaev:** Writing – review & editing, Writing – original draft, Supervision, Project administration, Formal analysis, Conceptualization.

## Data availability

The data that support the findings of this study are provided in the main text and figure and supplementary information.

## Conflict of interests

The authors declared no competing interests.
