# Supplementary material for: Clinical-molecular profiling of atypical GNAO1 patients: Novel pathogenic variants, unusual manifestations, and severe molecular dysfunction
Source: Genes Dis. 2025 Jan 9;12(5):101522. doi: 10.1016/j.gendis.2025.101522 (PMC12124604; doi:10.1016/j.gendis.2025.101522)
Supplement: Multimedia component 1 [file mmc1.pdf]

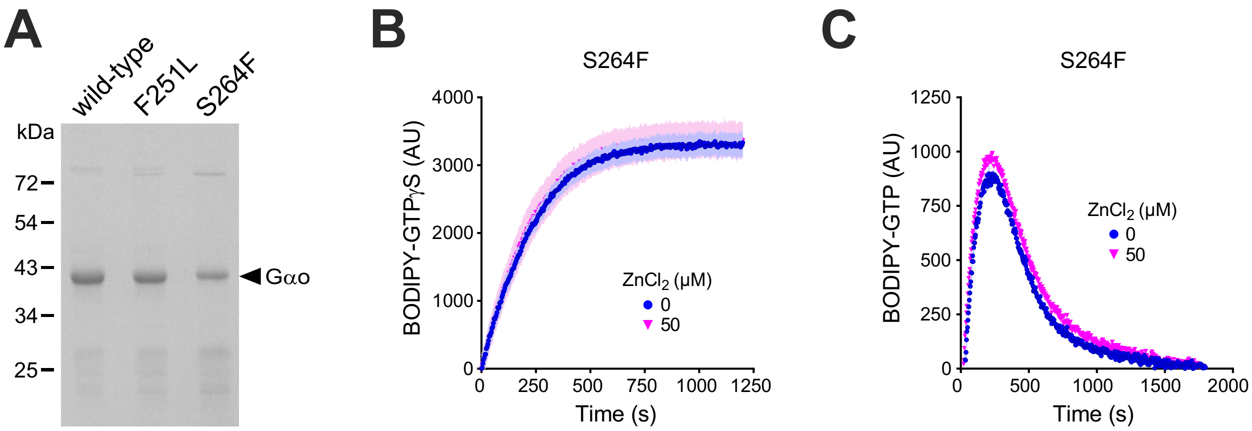

Figure S1. Sensitivity to Zn^{2+} of the His_6-Gαo S264F mutant. (A) Coomassie-stained gel analysis of recombinant His_6-tagged Gαo wild-type, F251L, and S264F. **(B, C)** The effect of 50 μM $ZnCl_2$ on BODIPY-GTPγS binding **(B)** and BODIPY-GTP hydrolysis **(C)** by Gαo S264F.
