# Supplementary material for: Clinical-molecular profiling of atypical GNAO1 patients: Novel pathogenic variants, unusual manifestations, and severe molecular dysfunction
Source: Genes Dis. 2025 Jan 9;12(5):101522. doi: 10.1016/j.gendis.2025.101522 (PMC12124604; doi:10.1016/j.gendis.2025.101522)
Supplement: Multimedia component 2 [file mmc2.pdf]

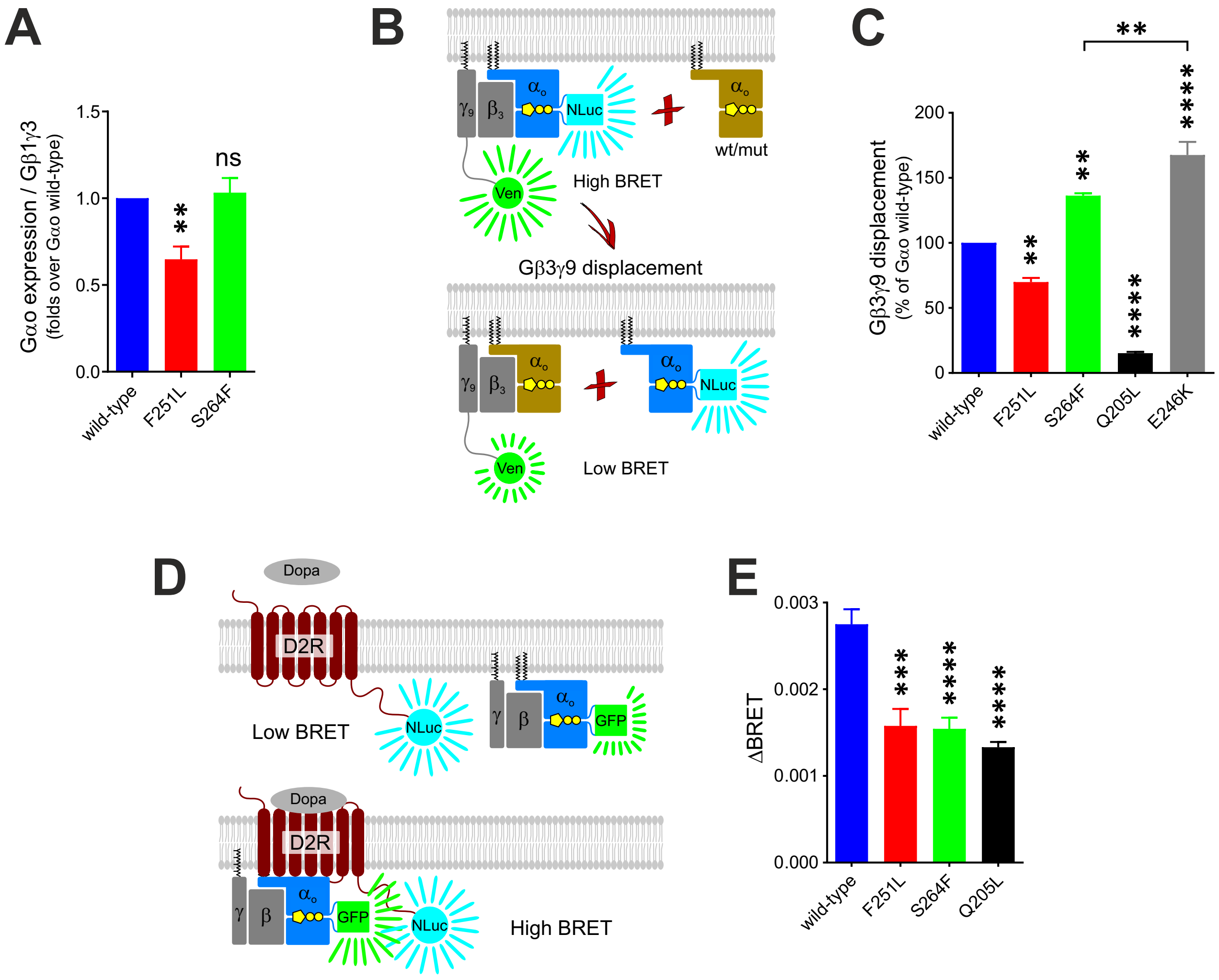

Figure S2. Gβ3γ9-binding and D2 dopamine receptor-coupling by Gαo F251L and S264F. **(A)** Quantification of the expression of Gαo wild-type, F251L, and S264F relative to the co-expression of Gβ1γ3 (*n*=5). **(B)** Scheme of the BRET-based Gβ3γ9 displacement assay. Gαo wild-type tagged with nano-luciferase (Gαo-NLuc) excites the Venus (Ven)-fusion of Gγ9 in the Gβ3γ9 heterodimer. **(C)** The ability of Gαo variants to displace Gαo-NLuc from Gβ3γ9 (reduction in the BRET signal) was quantified for Gαo wild-type, F251L, S264F, the constitutive active (non-pathogenic) Q205L mutant used as negative control, and the NEDIM-associated E246K variant used as control for an elevated Gβ3γ9-displacement (*n*=3). **(D)** Scheme of the BRET-based D2 dopamine receptor (D2R)-coupling assay. D2R tagged with nano-luciferase (D2R-NLuc) excites the GFP fusion of Gαo (Gαo-GFP). The basal BRET signal increased upon dopamine (Dopa) stimulation (ΔBRET). **(E)** Quantification of ΔBRET for Gαo wild-type, F251L, S264F, and Q205L (*n*=5). Data represent mean ± SEM. Data were analyzed by one-way ANOVA followed by Dunnett's multiple comparison test; ns is not significant, ***P*<0.01, ****P*<0.001, and *****P*<0.0001.
