# Supplementary material for: Clinical-molecular profiling of atypical GNAO1 patients: Novel pathogenic variants, unusual manifestations, and severe molecular dysfunction
Source: Genes Dis. 2025 Jan 9;12(5):101522. doi: 10.1016/j.gendis.2025.101522 (PMC12124604; doi:10.1016/j.gendis.2025.101522)
Supplement: Multimedia component 3 [file mmc3.pdf]

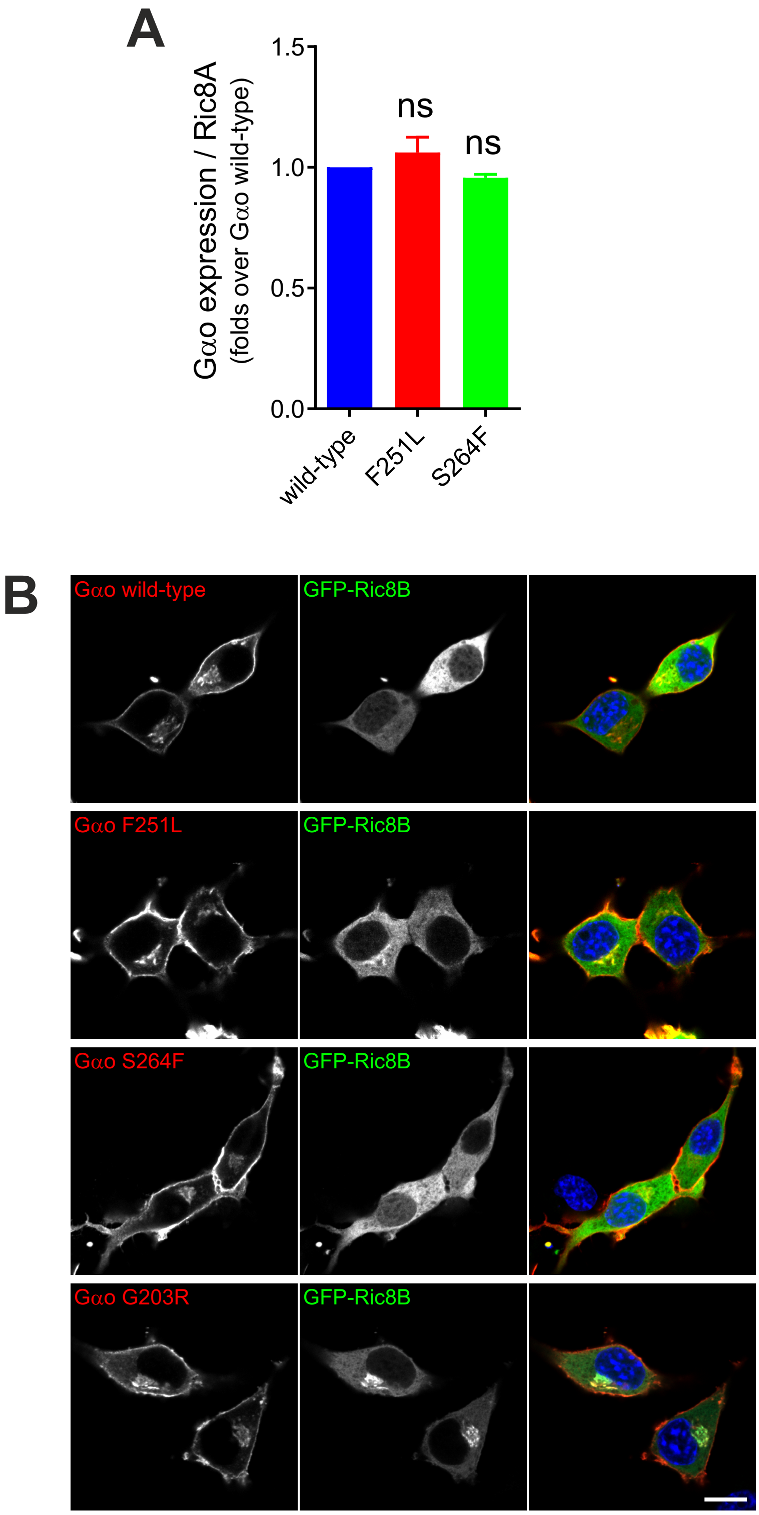

Figure S3. Golgi-relocalization of Ric8B by pathogenic Gαo mutants. (A) Quantification of the expression of Gαo wild-type, F251L, and S264F relative to Ric8A co-expression (n=5). Data represent mean ± SEM. Data were analyzed by one-way ANOVA followed by Dunnett's multiple comparison test; ns is not significant. **(B)** Confocal images of N2a cells co-expressing a GFP-fusion of Ric8B (GFP-Ric8B) alongside Gαo wild-type, F251L, S264F or the DEE17-linked G203R mutant used as control. Cells were immunostained against Gαo and nuclei were visualized with DAPI in blue. Scale bar, 10 μm.
