# Supplementary material for: Clinical-molecular profiling of atypical GNAO1 patients: Novel pathogenic variants, unusual manifestations, and severe molecular dysfunction
Source: Genes Dis. 2025 Jan 9;12(5):101522. doi: 10.1016/j.gendis.2025.101522 (PMC12124604; doi:10.1016/j.gendis.2025.101522)
Supplement: Multimedia component 4 [file mmc4.pdf]

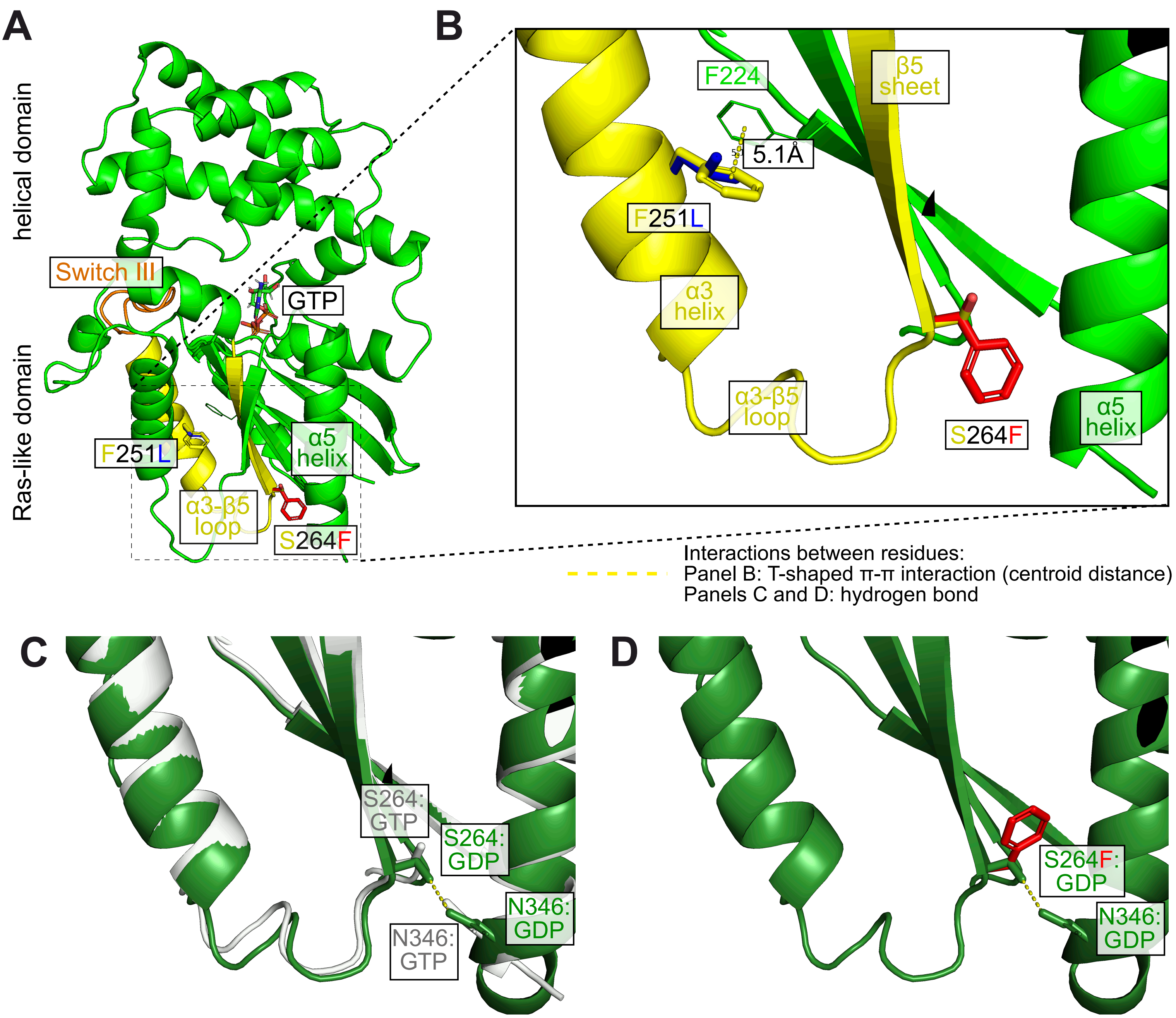

Figure S4. Structure homology modeling of Gαo F251L and S261F. (A) General view of the GTP-bound structure of Gαo with the backbone in green, the α3β5-loop is highlighted in yellow, and the Switch III domain in orange. The side chain of Leu replacing Phe at position 251 (F251L) is shown in blue, and the side chain of the Phe substituting the Ser at position 264 (S264F) is in red. **(B)** Magnification of the α3β5-loop illustrating the distance between the phenyl ring centroids of F251 and F224, and the T-shaped π-π aromatic interaction as a yellow dashed line. Note the loss of the interaction in the structure of the F251L variant. The short side chain of Ser at position 264 is not involved in any interactions in the GTP-loaded Gαo structure. **(C)** Alignment of the interface between the α3β5-loop and the α5-helix in the GTP- and GDP-bound states (white and dark green, respectively) of Gαo. Note the hydrogen bond (H-bond; yellow dashed line) between S264 and N346 of the α5-helix formed in the GDP-loaded Gαo. **(D)** Substitution of S264 by Phe is sterically possible but abolishes the H-bond with the α5-helix. Note that the relative positions of F251 and F224 are identical in both GDP- and GTP-bound states, not shown in panels **(C)** and **(D)**.
