# Supplementary material for: Clinical-molecular profiling of atypical GNAO1 patients: Novel pathogenic variants, unusual manifestations, and severe molecular dysfunction
Source: Genes Dis. 2025 Jan 9;12(5):101522. doi: 10.1016/j.gendis.2025.101522 (PMC12124604; doi:10.1016/j.gendis.2025.101522)
Supplement: Multimedia component 5 [file mmc5.docx]

**Material and methods**

## *Clinical and genetic evaluation*

Clinical information was collected from medical reports; caregivers gave informed consent for the study and publication of the video and patient-related information. The study was approved by the territorial ethics committee (approval #CET 80/24). The pathogenic variants were determined by epilepsy-focused gene panel (Illumina MiniSeq and kit Agilent Sureselect Custom) and whole exome sequencing (Agilent SureSelect Clinical Research Exome, Illumina PE 2x150) in two different diagnostic centers and the pathogenic variant was confirmed as *de novo* by Sanger sequencing of patients and patients’ both parents. Frequency analysis and pathogenicity prediction of the *GNAO1* variants were performed according to different in silico prediction programs (Table S1).

## *DNA Plasmids*

The plasmids for the non-tagged Gαo, His_6_-Gαo, Gαo-GFP (internal GFP-fusion at Gly92), GFP-Gβ1, GFP-Gγ3, GFP-RGS19, MannII-GFP, M2R-NLuc, D2R-NLuc, GFP-Ric8A, GFP-Ric8B were published earlier.^1-6^ Site-directed mutagenesis was used to introduce the F251L and S264F mutations into the non-tagged Gαo, His_6_-Gαo, and Gαo-GFP plasmids using the following oligonucleotide primers: F251L-f, 5’-CTCTCATGCTCCTCGACTCCATCTGTAACAACAAGTTC-3’; F251L-r, 5’-CAGATGGAGTCGAGGAGCATGAGAGACTCGTGCATGCGG-3’; S264F-f, 5’-CTTCATCGATACCTTCATCATTCTCTTCCTCAACAAGAAAG-3’; S264F-r, 5’-TGAGGAAGAGAATGATGAAGGTATCGATGAAGAACTTGTTG.

## *Recombinant Gαo*

The production and purification of recombinant Gαo was performed as previously described.^1;7^ Briefly, the Rosetta(DE3)pLysS *E. coli* strain was transformed with a pET23b-based plasmid encoding His_6_-tagged Gαo variants, and was grown at 37°C until OD_600_ reached ~0.6. Recombinant protein expression was induced with addition of 0.25 mM IPTG at 18°C overnight. Bacteria were harvested by centrifugation 3,500xg at 4°C and resuspended in TBS (20 mM Tris-HCl, pH 7.5, 150 mM NaCl) supplemented with 1 mM PMSF and 30 mM imidazole. Cells were disrupted in a High-Pressure Cell Press Homogenizer, the debris were removed by centrifugation at 15,000xg/15min/4°C. The supernatant was applied to the Ni^2+^ resin (Qiagen) overnight on a rotary shaker at 4°C. The Ni^2+^ resin was washed twice with 10x resin volumes of TBS supplemented with 10 mM imidazole. For the third wash, the washing buffer was supplemented with 3% glycerol, 10 mM MgCl_2_, 0.1 mM DTT, and 200 μM GDP. The Ni^2+^ resin was washed two more times with 10x resin volumes of the washing buffer. Proteins were then eluted with TBS containing 300 mM imidazole. Protein concentration was measured using the Bradford assay, and purity was analysed using SDS-PAGE followed by Coomassie staining.

## *GTP binding/hydrolysis assays*

The GTP binding and hydrolysis assay using BODIPY-GTP (Invitrogen) or BODIPY-GTPγS (Invitrogen) was performed as described.^1;7^ Gαo (1 μM) was diluted in the reaction buffer (TBS supplemented with 10 mM MgCl_2_ and 0.5% BSA) in black 384-well plates (Greiner), and BODIPY-GTP or BODIPY-GTPγS (1 μM) was added into the wells. ZnCl_2_ treatment was done as previously reported.^7;8^ Fluorescence measurements were performed at 28°C in a Tecan Infinite M200 PRO plate reader with excitation at 485 nm and emission at 530 nm. The GTP binding and hydrolysis data of Gαo were fitted to obtain the k_bind_ and k_hydr_ rate constants.^1;7^

## *Cell lines*

Mouse neuroblastoma Neuro-2a cells (N2a; ATCC) were cultured in Minimum Essential Medium, supplemented with 10% FCS, 2 mM L-glutamine, 1 mM pyruvate, and 1% penicillin-streptomycin. Human HEK293T cells (ATCC) in Dulbecco’s modified Eagle’s medium, supplemented with 10% FCS, 2 mM L-glutamine, and 1% penicillin-streptomycin. Cells were grown at 37°C and 5% CO_2_. Plasmid transfections were performed with X-tremeGENE-HP (Roche) or TransIT-2020 (Mirus).

## *Immunofluorescence and microscopy*

Localization of Gαo variants in N2a cells was studied by immunofluorescence and microscopy as reported earlier.^5;6^ Briefly, N2a cells (1.5x105 cells/well) were seeded on culture plates for 24 h before transfection, then were transfected for 7 h, trypsinized and seeded on poly-L-lysine-coated coverslips in complete MEM for 15-17 h before fixation. Immunostaining was done with an antibody (Ab) against Gαo (sc-13532; Santa Cruz Biotechnology), and DAPI (Sigma-Aldrich) was used to label nuclei. The secondary anti-Mouse AlexaFluor594-conjugated (715-585-150) was from Jackson ImmunoResearch. Cells were recorded in a LSM800 confocal microscope using the ZEN 2.3 software (Zeiss).

## *Co-Immunoprecipitations*

HEK293T (2x10^5^ cells /well) were seeded on plates and cultured for 48h before co-transfection. Co-Immunoprecipitation (co-IP) of GFP-tagged constructs was performed using a recombinant GST-tagged nanobody against GFP.^3;5^ IP of GFP fusions and co-IP of Gαo variants were determined by Western blot using Abs against GFP (PABG1; Proteintech) and Gαo (sc-393874; Santa Cruz Biotechnology). Secondary anti-mouse horseradish peroxidase (HRP)-conjugated (115-035-146), and anti-rabbit HRP-conjugated (111-035-144) were from Jackson ImmunoResearch. Quantification of blots was done using ImageJ-v1.54f, and images were edited using EvolutionCapt-v18.11 (Vilber).

## *Bioluminescence Resonance Energy Transfer (BRET) assays*

The BRET-based Gβ3γ9 displacement and GPCR-coupling assays were performed as previously reported.^4-6^ HEK293T cells (48,000 cells/well) were seeded in 48-well plates for 24 h before transfection. For Gβ3γ9 displacement assay, cells were co-transfected with the Go1-CASE plasmid^9^ and non-tagged Gαo or empty pcDNA3.1 (1:3 ratio). For the GPCR-coupling using the M2 muscarinic acetylcholine receptor (M2R) and the D2 dopamine receptor (D2R), cells were co-transfected with the M2R-NLuc or D2R-NLuc plasmids and Gαo-GFP or GFP (1:5 ratio). Cells were seeded at 12,000 cells/well in transparent-bottom black 384-well plates 24 h after transfection. The next day, the medium was replaced with 10 μl of PBS before BRET measurement using a Tecan Infinite plate reader. For steady state BRET assays (Gβ3γ9 displacement), furimazine (10 μM) was injected immediately before measurement. For kinetic BRET assays, furimazine was added before measurement and an agonist solution (10 μM acetylcholine for M2R or 10 μM dopamine for D2R) was injected sequentially.

The BRET signal was determined by calculating the ratio of the light emitted by the GFP- or Venus-tagged protein over the light emitted by the NLuc-fusion protein using the built-in NanoBRET filter system. For GPCR-coupling, the average baseline value (basal BRET ratio) recorded prior to agonist stimulation was subtracted from the experimental BRET signal values to generate ΔBRET.

## *3D structure modelling*

Homology modelling of the Gαo variants was performed by the SWISS-MODEL server using default settings. The resulting models were visualized and analyzed using PyMol. The initial molecular model was constructed in an energy-minimized form using ProMod3, which is integrated into the OpenStructure suite. The templates for the homology modelling were Gαi3 for the GTP-bound (8gvx) and Gαi1 for the GDP-bound (1bof) structures available at RCSB (rcsb.org). Subsequent optimization of the side chain rotamers was performed using TreePack and SCWRL4 for energy minimization. Further refinement of the model involved energy minimization protocols via OpenMM using the CHARMM22/CMAP force field. Ligand positioning was adopted from the template structure, provided there were no steric hindrances. Potential steric hindrances that could arise from the introduction of the mutation were resolved by energy minimization. Thus, the free ΔΔG energy differences of the minimized state for mutants and wild-type structures were estimated to be below the 1 kcal/mol threshold, indicating compatibility with the overall stability of the protein. Moreover, no significant alteration to the backbone was required to accommodate the modified residues, and their overall geometry satisfied the criteria for allowed Ramachandran conformations.

Interestingly, Knight *et al*.^10^ recently assessed the impact of two *GNAO1* mutations – c.748C>T; p.L250F and c.754G>A;p.D252N – that target the α3-helix of Gαo as the F251L variant we described here. L250F and D252N were classified into two opposing groups based on their impact on the G-R-E triad (composed of residues Gly204, R209, and E246 in Gαo), which plays a pivotal role in the GTP-induced structural rearrangements in Gα-subunits.^11^ The fact that the L250F substitution induced a significant defect in GPCR-signaling and D252N behaves as Gαo wild-type,^10^ suggests that the nature of the substitution holds greater significance than the position affected. Additionally, both L250F and D252N variants were evaluated for thermostability and exhibited only minor deviations from the wild-type protein, indicating that the region has a relatively limited influence on the overall structure of Gαo. Unfortunately, the phenotypes in the Patients carrying the L250F and D252N variants were not reported in Knight *et al*., denying any possible comparison with our Patient. In the ClinVar database, however, L250F and D252N are classified as “conflicting classifications of pathogenicity” and “likely benign”, respectively, somewhat agreeing with their very mild biochemical/cellular defects.

On the other hand, the S264F mutation appears as the first of its kind, as additional *GNAO1* mutations targeting the β5-sheet have not been reported thus far. It is worth noticing, however, a *GNAO1* mutation that introduces a deletion of I344 (c.1030_1032delATT;p.I344del), which has been linked to a rather mild phenotype.^4;12^ Similar to S264F, the recombinant Gαo I344del presented only minor biochemical defects and showed a reduced coupling to GPCRs,^4^ although the latter defect is more pronounced for I344del. Our unpublished observations indicate that Gαo I344del does not gain the neomorphic Ric8A/B interactions, adding another layer of similarity with S264F. I344 lays within the α5-helix nearby N346, which in turn is predicted to form an H-bond with S264 in the GDP-bound state of Gαo. Thus, these structural analyses suggests that *GNAO1* mutations that affect GPCR-coupling by targeting the β5/α5 interface – without severely impacting the overall Gαo structure – might represent a subgroup within the milder end of the spectrum of *GNAO1*-related disorders.

# Detailed Clinical Case Reports

Patient 1 is a 13-year-old boy born to healthy unrelated parents with no prior family history of neurologic disorders. During pregnancy, the mother was taking acetylsalicylic acid for a history of multiple miscarriages, and she was on a diet for the treatment of gestational diabetes. He was born by planned caesarean section at 39 weeks for breech presentation, birth weight 3240 g. No perinatal problems were reported.

At 6 months of age the patient had a brief episode of hypotonia and ocular revulsion during a febrile episode. During the first year, axial hypotonia and delayed psychomotor development were reported: sitting at 12 months, standing at 18 months and independent walking at 20 months. The patient lacked expressive language and contextual understanding. Stereotyped movements of the upper extremities during excitation, selective feeding and restricted and repetitive interests were also described. At the age of 12, he experienced two epileptic seizures characterized by head deviation and secondary generalized tonic-clonic seizures. He was treated with valproic acid with complete seizure control. Currently, he presents with a normal neurological examination without movement disorders and dominated by severe intellectual disability with absence of speech and autism- like social- emotional and behavioral difficulties (Video S1, Segment 1).

Brain MRI was normal; repeated EEG assessment revealed epileptic abnormalities in the right central or anterior regions. A heterozygous *de novo GNAO1* variant NM_020988.3: c.751T>C;p.Phe251Leu was identified by whole-exome sequencing (Table S1). This variant has not been reported in the literature and absent in the general population (MAF0), and resulted as probably damaging using in silico analysis with established predictive tools (PolyPhen-2 and SIFT).

Patient 2 is a 16-month-old girl born to healthy unrelated parents with no history of neurologic disorders. She was born at term after an uncomplicated pregnancy with induced vaginal delivery for premature rupture of membranes, with an Apgar index of 9-10 and the weight of 3447 g; the maternal vaginal swab was positive for streptococcus.

In the first hours of life, she presented apnoeic episodes with desaturation. Antibiotic therapy was initiated on suspicion of early sepsis; she required respiratory support for 12 hours, which was discontinued due to remission of this symptomatology. After 48 hours, episodes of staring, apnoea, cyanosis and desaturation up to 50% occurred again, without rhythmic motor phenomena or associated automatisms and without a clear reduction in vigilance. These episodes lasted about 1 minute and resolved after repeated tactile stimulation, but oxygen therapy was always required. The baby was transferred to the neonatal intensive care unit, where phenobarbital therapy was started with complete remission. The EEG showed discrete organization and inconstant asymmetry of electrical activity, with rare sharp waves in short sequences mixed with monomorphic theta in centrotemporal regions. Brain MRI was normal.

After an initial hypotonia, subtle hypertonia of the scapular girdle and upper limbs, sialorrhea, and rhythmic protrusion of the tongue were reported. These symptoms gradually decreased during the first year of life. Occasional startle reactions were observed in the first few months.

Overall, psychomotor development was broadly normal (head control at 3 months, independent sitting at 7-8 months without lateral parachutes, crawling at 10 months, independent walking at 15 months; first words around one year with subsequent poor development). Minimal bradykinesia and difficulties in motor organization were immediately noted after age 3 months (Video S1, segments 2 and 3). The patient was followed up from the first few months with an intensive cycle of physiotherapy and speech therapy.

Development was assessed at 7 and 11 months of age using the Griffiths Mental Development Scale (GMDS). The Developmental Quotient (DQ) was 90 and 83 and 81 at 7 and 11 and 16 months respectively, with mild difficulties in the scales assessing eye-hand coordination and personal-social-emotional skill

At present, her neurological examination is characterized by a mild rigid hypokinetic syndrome (Video S1, Segments 4 and 5). No other movement disorders were noted. She continues to be seizure free. Phenobarbital therapy is being tapered with no clinical change. Rare and brief episodes of bradycardia (up to HR 60 bpm without change in saturation) during nighttime sleep were noted at home, but cardiologic evaluation with Holter ECG and ultrasound showed normal results.

A *de novo* variant of *GNAO1* was identified through next-generation sequencing panel for early epileptic encephalopathies: NM_020988.3: c.791C>T;p.Ser264Phe. This variant has not been reported in the literature, but its absence in the general population (MAF0), location in a relatively conserved amino acid residue, and in silico analysis with established predictive tools (PolyPhen-2 and SIFT) suggest that it may be pathogenic (Table S1).

## Video S1

Segment 1: Patient 1 at age 13 during spontaneous motor activity that appears normal overall and without evidence of movement disorder.

Segment 2: Patient 2 at age 8 months. The patient in supine and prone position presents a slow spontaneous motor activity with a poor repertoire.

Segment 3: During grasping, clumsiness and abnormal posture of both hands are observed

Segments 4 and 5: Patient 2 at age 17 months. The child has acquired autonomous walking and is able to perform postural changes independently. However, the child demonstrates some bradykinesia and dystonic posturing of the foot.

**Table S1. Genetic evaluation of *GNAO1* variants.**

| **Patient** | **GNAO1 variant** | | **CADD score** | **SIFT** | **Poly Phen 2**  **(Hum Var)** | **Mutation Tester** | **GnomAD frequency** |
| --- | --- | --- | --- | --- | --- | --- | --- |
|  | **cDNA change** | **Amino acid change** |  |  |  |  |  |
| Patient 1 | c.751T>C | p.Phe251Leu  (F251L) | 28.4 | Deleterious (0.02) | Probably Damaging (1.000) | Disease causing  (0.999) | Absent |
| Patient 2 | c.791C>T | p.Ser264Phe  (S264F) | 32 | Deleterious (0) | Probably Damaging (1.000) | Disease causing  (0.999) | Absent |

In silico tools used to predict variant pathogenicity included Combined Annotation Dependent Depletion (CADD,cadd.gs.washington.edu/snv), Sorting Intolerant from Tolerant (SIFT, Genome Institute of Singapore, <http://sift.bii.a-star.edu.sg/>), Polymorphism Phenotyping v2 (PolyPhen-2) (<http://genetics.bwh.harvard.edu/pph2/index.shtml>), MutationTaster (<https://www.mutationtaster.org/>), gnomAD (<https://gnomad.broadinstitute.org/>). For all cases and the further genetic investigations, the reference sequence NM_020988.3 was used.

# References

1. Lin C, Koval A, Tishchenko S, et al. Double suppression of the Galpha protein activity by RGS proteins. *Mol Cell.* 2014;53(4):663-671.

2. Solis GP, Bilousov O, Koval A, Luchtenborg AM, Lin C, Katanaev VL. Golgi-Resident Galphao Promotes Protrusive Membrane Dynamics. *Cell.* 2017;170(5):939-955 e924.

3. Solis GP, Kozhanova TV, Koval A, et al. Pediatric Encephalopathy: Clinical, Biochemical and Cellular Insights into the Role of Gln52 of GNAO1 and GNAI1 for the Dominant Disease. *Cells.* 2021;10(10):2749.

4. Lasa-Aranzasti A, Larasati YA, da Silva Cardoso J, et al. Clinical and Molecular Profiling in GNAO1 Permits Phenotype-Genotype Correlation. *Mov Disord.* 2024.

5. Solis GP, Koval A, Valnohova J, Kazemzadeh A, Savitsky M, Katanaev VL. Neomorphic Galphao mutations gain interaction with Ric8 proteins in GNAO1 encephalopathies. *J Clin Invest.* 2024;134(15).

6. Solis GP, Larasati YA, Thiel M, Koval A, Koy A, Katanaev VL. GNAO1 Mutations Affecting the N-Terminal alpha-Helix of Galphao Lead to Parkinsonism. *Mov Disord.* 2024;39(3):601-606.

7. Larasati YA, Savitsky M, Koval A, Solis GP, Valnohova J, Katanaev VL. Restoration of the GTPase activity and cellular interactions of Galpha(o) mutants by Zn(2+) in GNAO1 encephalopathy models. *Sci Adv.* 2022;8(40):eabn9350.

8. Larasati YA, Thiel M, Koval A, Silachev DN, Koy A, Katanaev VL. Zinc for GNAO1 encephalopathy: Preclinical profiling and a clinical case. *Med.* 2024.

9. Schihada H, Shekhani R, Schulte G. Quantitative assessment of constitutive G protein-coupled receptor activity with BRET-based G protein biosensors. *Sci Signal.* 2021;14(699):eabf1653.

10. Knight KM, Obarow EG, Wei W, et al. Molecular annotation of G protein variants in a neurological disorder. *Cell Rep.* 2023;42(12):113462.

11. Knight KM, Ghosh S, Campbell SL, et al. A universal allosteric mechanism for G protein activation. *Mol Cell.* 2021;81(7):1384-1396 e1386.

12. Kelly M, Park M, Mihalek I, et al. Spectrum of neurodevelopmental disease associated with the GNAO1 guanosine triphosphate-binding region. *Epilepsia.* 2019;60(3):406-418.
